# A qualitative comparison of how older breast cancer survivors process treatment information regarding endocrine therapy

**DOI:** 10.1371/journal.pone.0210972

**Published:** 2019-01-31

**Authors:** Huibrie C. Pieters, Emily Green, Sally Khakshooy, Miriam Sleven, Annette L. Stanton

**Affiliations:** 1 School of Nursing, University of California at Los Angeles, Los Angeles, United States of America; 2 Cedars Sinai Medical Center, Los Angeles, United States of America; 3 Torrance Memorial Medical Center, Torrance, California, United States of America; 4 Departments of Psychology and Psychiatry & Biobehavioral Sciences, University of California, Los Angeles, United States of America; Brown University, UNITED STATES

## Abstract

**Background:**

It remains unclear how information about aromatase inhibitors (AI) impacts women’s decision-making about persistence with endocrine therapy.

**Purpose:**

To describe and compare how women treated for primary early stage breast cancer either persisting or not persisting with an AI received, interpreted, and acted upon AI-related information.

**Design:**

Thematic analysis was used to sort and compare the data into the most salient themes.

**Participants:**

Women (N = 54; 27 persisting, 27 not persisting with an AI) aged 65–93 years took part in qualitative interviews.

**Results:**

Women in both subgroups described information similarly in terms of its value, volume, type, and source. Aspects of AI-related information that either differed between the subgroups or were misunderstood by one or both subgroups included: (1) knowledge of AI or tamoxifen prior to cancer diagnosis, (2) use of online resources, (3) misconceptions about estrogen, hormone replacement therapies and AI-related symptoms, and (4) risk perception and the meaning and use of recurrence statistics such as Oncotype DX.

**Conclusions:**

Persisters and nonpersisters were similar in their desire for more information about potential side effects and symptom management at AI prescription and subsequent appointments. Differences included how information was obtained and interpreted. Interactive discussion questions are shared that can incorporate these findings into clinical settings.

## Introduction

In the US population, breast cancer is increasingly common in older women, with the median age of diagnosis at 61 years [1]. The majority of breast cancers (60%–70%) express the estrogen or progesterone receptor or both. Therefore, following primary treatments, an endocrine therapy such as an aromatase inhibitor (AI) or tamoxifen is the standard of care for postmenopausal women with hormone receptor–positive breast cancer. In 2000, the National Institutes of Health consensus conference recommended 5 years of adjuvant tamoxifen for women with hormone receptor-positive tumors larger than 1 cm [2]. Ensuing studies recommended the use of AIs for 5 years in postmenopausal women, with further studies suggesting endocrine therapy for up to 10 years in certain situations [3]. However, despite the efficacy of AIs in reducing the risk of cancer recurrence, rates of discontinuation increase over time from 90% persisting at 1 year to only 50% at 5 years [4]. A systematic review [5] has shown a mean of only 79% at 1 year and 56% at 5 years. Our work is focused on persistence, defined as the duration from initiation to discontinuation of therapy [6], in contrast to adherence which reflects taking the correct dose according to frequency [7]. Nonpersistence rates appear to be especially high in older adults [6, 8–12], though findings are mixed [5].

While medication adherence is widely studied, factors that impact nonpersistence and ways to support medication adherence to AIs remain poorly understood. Literature reviews have focused on adherence to endocrine therapies and highlighted the complex dimensions that contribute to early discontinuation of AIs. In a recent review [13] the following were concluded to be primary reasons for discontinuation: lack of knowledge about the role and benefits of endocrine therapy, uncontrolled adverse effects, concerns about rare but serious toxicities, cost of medications, distrust of health system, poor communication with medical staff and a lack of perceived risk for recurrence. A recent systematic review found many similar factors related to persistence, yet overall, findings on modifiable and psychosocial factors influencing adherence were inconsistent [5].

Reviews of approaches to improve adherence to a variety of medication regimens have identified strategies such as patient education including providing written information, discussing side effects and assessing a patient’s understanding of the treatment, and patient support including providing ready access to health care professionals, side effect management, and treatment monitoring [5, 14–16]. Specific to endocrine therapy, additional interventions recommended to increase adherence and persistence include improving patient-provider communication, patients’ understanding of treatment benefit, and side effect management [13]. These areas require attention at both initial treatment discussions and during ongoing follow up.

The randomized controlled trials aimed at improving adherence with AIs all have tested informational and educational interventions [17–21]. Despite targeting information and consultation related to treatment and treatment concerns, all the trials to date demonstrated no significant improvement of adherence [22]. Although information, education, knowledge, understanding of treatment importance and side effect management were identified as critical to support adherence, clinical trials to date have failed to improve adherence.

At this time, the complex relationship of how information is received during the treatment trajectory and how it may impact decision-making processes regarding adherence to endocrine therapy remains unclear. Therefore, as part of a larger study to describe the age-related perspective of how, in their own words, the underrepresented and high risk population of older survivors of primary, loco-regional breast cancer decided to persist or prematurely stop an AI, the present study addressed the nuances of treatment-related information. Aims were to 1) describe how women received, interpreted, and acted upon information about the role of AIs, and 2) compare how women either persisting or not persisting with an AI at the time of interview differed and were similar in the ways they viewed, used or acted upon information related to their AI treatment. Based on the data, a potential outcome was to develop materials for practitioners to facilitate communication about AIs.

## Methods

Grounded theory informed by a constructivist worldview guided all aspects of this research. The philosophical tenets that underlie constructivist grounded theory, symbolic interactionism and pragmatism, are deeply steeped in how knowledge is perceived, understood and retained in ongoing interactions with self and others [23].

### Participants

Eligible women were at least 65 years old, started an AI for loco-regional (Stage I, II or III) breast cancer 4–36 months prior to enrollment and were in charge of taking their own medications. Two groups were recruited: women who self-reported continuing with the endocrine therapy and women who had intentionally stopped within the past 15 months. The time limit of 15 months was chosen to improve the accuracy and quality of participant reflection.

### Procedures

Approval of the study was received from the South General Institutional Review Board of UCLA (Protocol number 13–000526), the Institutional Review Board of Torrance Memorial Medical Center (Protocol number 2014.11.1), and the California Health and Human Services Agency’s Committee for the Protection of Human Subjects for the Cancer Registry of Central California (Protocol number 14-06-1627). Once approvals were received a mailed invitation was sent to prospective participants from three cancer registries in Southern California. Recruitment flyers were also placed in oncology waiting areas of hospitals and community settings such as cancer support organizations. Recruitment took place from October 2013 to April 2016, with the subgroup of non-persisters proving more challenging to recruit. Data collection continued until no new properties and dimensions were identified and the analysis showed nuanced themes. As it happened, both subgroups saturated with equal numbers (n = 27 each).

Following written consent, data were collected with an in-person interview and from medical records. The first author, who is highly experienced in qualitative research, conducted the interviews in a private place of the woman’s choosing. Except for an introductory call to confirm the place and time of the interview, participants did not know the interviewer prior to the meeting. Semi-structured questions informed by existing literature and our clinical experience guided the intensive interviews in a way that allowed each participant to focus on what mattered most to her. An iterative inductive process was used where data was analyzed from the start of data collection and the analysis informed future interviews. (See Table 1 for exemplars.) Interviews were audio-recorded and the subsequent professional transcriptions were de-identified and checked for accuracy.

**Table 1:** Examples of conversational interview questions and potential follow-up prompts.

How did you get information on the (name that the participant used for the AI)? Follow up question: Some women also searched online for information. How did that work for you?
Overall, what do you think about the quality of the information that you received? Follow up question: What about the quality was helpful for someone in your position or what was less helpful? Follow up question: How do you feel about the amount of information that you received from your oncologist?
How would you describe the communication that you had with your oncologist after you finished the radiation treatment (or whichever primary treatment was received last)? (If favorable) Follow up: What did the doctor do or provide that made you feel confident in taking (the name that the woman gives the AI)? (If disappointing) Follow up: What improvements can be made so that patients such as yourself can be better prepared to continue the (name of AI)?
What motivated you to start the (name)? Follow up: What is your understanding of why the medication was prescribed for you?
As you see it, was your oncologist open to discuss your questions /concerns about your (medication name) usage? (If no) Follow up: How do you understand this? (If no) Follow up: Did you turn to someone else instead?
Did you seek information from anyone else? (If primary care provider) Follow up: What about your primary doctor made him/ her a good source of information for you? (If another survivor) Follow up: What about connecting with this other woman/ friend who had also been treated helped you obtain more information? (If she received conflicting information) Follow-up question: So what Dr. (name) said and what you heard from (e.g. name of primary) was different is some ways. How was that for you?
Who gave you the most helpful or useful information regarding AIs? Follow up: What about this information was particularly helpful for you?
Please tell me about your decision to continue (or stop) the (medication name). Non-persisters Follow up: Please remember that the purpose of our conversation is not to motivate you to restart the medication. So, in all honesty, please tell me the reasons that led to your decision to stop the (medication name)? How do you feel about your decision to stop? Persisters Follow up: What motivates you to continue with the (medication name)?

### Data analysis

In this thematic analysis [24], three researchers (HCP, EG and MS) carefully read each transcript to familiarize themselves with the data. Subsequently we completed repeated rounds of systematic substantive coding using abductive reasoning and iterative processes. Initial coding was done line by line [23]. Differences in opinions and inconsistencies as we generated the initial codes were easily resolved during the regular meetings of the three-person analytic team. Then, across participants, we sorted, compared and identified codes. We searched and identified unique areas of similarities and differences between the women who persisted and those who had stopped. We further sorted the data into themes based on similarities and differences. Subsequently we initiated a comparative analysis that involved displaying the data in a visual format to allow for a systematic comparison of the two subgroups using tables in Microsoft Word and Excel [25]. The relatively large sample for a qualitative study (N = 54) and extensive interviews (mean duration = 97 minutes) with detailed data allowed for such a comparison. Observational field notes, memo-writing and diagramming in keeping with grounded theory techniques [23] were also used to further develop themes. Atlas-ti was used to organize the data.

During the analysis, it came to the researchers’ attention that the two subgroups were not as distinct as had been expected. At least three women from the persister subgroup described the intention to stop while five women in the non-persister subgroup were open to the possibility of restarting the AI. Therefore, while “current persister” and “current non-persister” describe the fluidity of the subgroups, we abbreviated to “persisters” and “non-persisters” for the sake of simplicity. In our sample, the AIs were prescribed, managed and discussed by the participants’ medical oncologist. The majority of participants, 98%, received follow-up care from a medical oncologist or nurse practitioner in the same practice who continued to monitor their status whether on or off the AI.

## Results

The 54 women were a mean age of 71.9 years (range = 65–93) at diagnosis. Half the sample was persisting with an AI at the time of interview, and half had prematurely discontinued the treatment. Non-persistence was intentional and no participant identified a comorbid condition, medication management, or financial barriers as a reason to discontinue endocrine therapy. Most participants self-identified as non-Hispanic white (n = 44) and were at least college graduates (n = 38). (See Tables 2 and 3 for sample characteristics).

**Table 2:** Demographic characteristics.

Characteristic		AI Persisters (n = 27)	AI Non-persisters (n = 27)	Total sample (N = 54)
*Age at interview (years)	Mean	73.3	73.6	73.4
	Range	66–91	65–94	66–94
*Months from diagnosis to interview	Mean	18	21.2	19.6
	Range	9.3–38	8.2–44.8	8.2–44.8
**Self-identified race/ethnicity	White	19	25	44
	Latina	2	1	3
	Japanese	3	0	3
	Chinese	1	1	2
	Korean	1	0	1
	African American	1	0	1
**Marital status	Married	12	12	24
	Never Married	1	1	2
	Widowed	9	7	16
	Divorced	4	6	10
	Separated	1	1	2
**Education completed	High school graduate	7	2	9
	Some college	2	5	7
	College graduat	9	10	19
	Some graduate school	3	2	5
	Graduated from graduate school	6	8	14
**Annual household income	<$20,999	1	1	2
	$21,000-$40,999	5	9	14
	$41,000-$60,999	3	5	8
	$61,000-$80,999	8	5	13
	$81,000-$100,999	4	1	5
	>$101,000	6	6	12
**Living situation	With Spouse	12	12	24
	Alone	13	10	23
	Other	2	5	7
**Mini-Mental Status Examination	Mean	29	28.7	29.4
	Range	28–30	24–30	24–30
**Used online sources for AI-related information	Yes	14	22	36
	No	9	4	13
	Not asked	4	1	5
**Aware of AI prior to diagnosis or prescription	Knew of AI	5	10	15
	No prior knowledge	21	14	35
	Not asked	1	3	4
**Aware of tamoxifen prior to diagnosis or prescription	Yes	9	16	25
	No	11	5	16
	Not asked	7	6	13
**Knew someone with taking/had taken tamoxifen or AI	Yes	11	19	30
	No	11	0	11
	Not asked	5	8	13

*Source: Medical record

**Source: Self-report

**Table 3:** Clinical characteristics.

Characteristic		AI Persisters (n = 27)	AI Non-persisters (n = 27)	Total sample (N = 54)
*Breast cancer stage	I	15	20	35
	II	9	6	15
	III	3	1	4
**Primary treatments received	Lumpectomy	19	21	40
	Lateral mastectomy	5	5	10
	Double mastectomy	3	1	4
	Radiotherapy	20	17	37
	Chemotherapy	7	2	9
*Charlson comorbidity index	Mean	3.3	2.8	3.1
	Range	2–8	2–6	2–8
*Number of positive lymph nodes removed		5	3	8
**Setting of where treatment was received	NCI-designated comprehensive cancer center	15	6	21
	Private practice	3	12	15
	Community medical center	6	4	10
	HMO	2	5	7
	Clinic	1	0	1
*Oncotype DX documented in medical record		8	11	19
**Ever taken HRT	Yes	19	21	40
	No	8	6	14
**Stopped HRT at time of diagnosis		5	12	17
**Stopped HRT before diagnosis		14	9	23
*Mean duration of treatment on endocrine therapy		13.3 months	11.1 months	12 months
*Months between last date endocrine therapy taken to interview date	Mean		6.4	
	Range		0.6–14.6	

*Source: Medical record

**Source: Self-report

### Similarities across persisters and non-persisters

Overall, the information experiences and needs of women continuing AIs and those who had prematurely discontinued were largely similar. Women in both subgroups described information similarly in terms of its value, desired volume and what type of information they had or wanted to have. Both persisters and non-persisters valued information and many wanted to make decisions based on information. One participant represented herself as someone who “got as much information as I can” and another identified as “the type who wants to know everything”.

Women in both subgroups reported receiving a range of information on AIs. Many recounted specific details of the mechanism of the medications, how their type of cancer was related to the AI, and potential side effects. Less known, across subgroups, were self-management tips and the possibility of changing to a second AI if there was a problem. Preferences varied widely across subgroups regarding satisfaction with the volume of information received, ranging from being fully satisfied to feeling on her own looking for answers and wishing she had received much more from her medical team. In terms of receiving information, both subgroups described receiving conflicting input from healthcare providers such as the non-persister who recalled that her long-term gynecologist said he thought "it was foolish for me to be taking it (AI)" after the prescribing oncologist encouraged her to take the AI. In addition, a number of women in both subgroups reported overall very poor recall of the information they received about AIs, ranging from no memory of certain aspects being reviewed to substantial misunderstandings or incorrect information.

Regarding the type of information, in addition to wanting to know about recurrence and general cancer prevention, most women in both subgroups wanted to know about side effects and how to manage them. Some women described how, had they known what potential side effects were coming, they would have been able to understand that symptoms they experienced were related to the AI. Many participants felt under-prepared and one woman specifically compared receiving information about endocrine therapy to receiving information about chemotherapy and radiation saying, “I think up front you should also be told. Up front, you're told about the chemo and how you will be sick from it. And you're told about the radiation and how it's not going to affect you very much. And I would like to have known about the pill and all of its effects, but I did not …we should be told it’s a mean little pill.” However, women across both subgroups also reported adamantly not wanting to hear or seek out information on side effects in order to not, as one woman put it, “psych myself out”.

Pertaining to the sources of information, women referenced their oncology providers, other medical providers, peer survivors and friends, and online and additional outside resources. All participants described medical oncologists as the expert source of treatment-related information. However, information from other people, online resources and the oncologist all competed for the women’s understanding of their AI-treatment. Women in both subgroups described the frequency of interactions with their providers, the quality of information received, their actual or preferred communication styles, and their tendency to trust and follow provider recommendations. Both subgroups looked to peer survivors and female friends for practical tips and emotional support. Women who discontinued also leaned on this group to validate their experience and help position and contextualize their decision to discontinue the therapy. Both subgroups had women who used, albeit comparatively minimally, additional sources of information such as pharmacy inserts, breast cancer navigators, and other trusted physicians.

### Differences between persisters and non-persisters

In contrast to the experiences and information needs being similar across subgroups, four aspects of AI-related information stood out as either misunderstood or different by one or both subgroups: (a) knowledge of AI and/or tamoxifen prior to breast cancer diagnosis, (b) use of online resources for AI-related information gathering, (c) misconceptions about estrogen, hormone replacement therapies and AI-related symptoms, and (d) the meaning and use of recurrence statistics.

#### Knowledge of endocrine therapies prior to breast cancer diagnosis

In the interviews, women were asked about their knowledge of AIs and tamoxifen prior to their own cancer diagnosis and treatment. Non-persisters were more familiar with endocrine therapies than were persisters. Of the 27 non-persisters, 10 stated they knew specifically about AIs prior to diagnosis or receiving the prescription and 16 were familiar with tamoxifen only. In contrast, only 5 persisters had heard about AIs and 9 knew about tamoxifen only. Nineteen non-persisters reported they had a friend or family member who was taking or had taken some kind of endocrine therapy compared to 11 persisters. Therefore, women who discontinued their endocrine therapies prematurely were more likely to have prior knowledge of AIs. Across both subgroups, of the women who had heard of endocrine therapies, most reported that they did not know details such as duration of treatment, side effects, or the exact names of the medications. (See supporting quotes in Table 4 with reference to Results in parenthesis.)

**Table 4:** **Obtaining and understanding information regarding endocrine therapy among persisters and non-persisters:** A range of exemplars.

	AI persisters (n = 27)	AI non-persisters (n = 27)
Knowledge of tamoxifen and/or AI prior to breast cancer diagnosis	“I had not heard of that (AI). I thought I would be through after (surgery and radiation). It’s a little bit disappointing. I was upset that it’s going to drag on and on and on, but I do it. And they were so kind to me helping me get everything over with quickly, quickly, quickly and then I find out, no.” (Unfamiliar with AI prior to diagnosis) “You know, I never heard of any of this before. I never even knew D (friend) used to take a pill for some time. You just don't talk about those things when you don't have cancer. You just don't think about it at all.” (Unfamiliar with endocrine therapies prior to diagnosis) “I knew that there were people who'd had like a lumpectomy and were on tamoxifen. And I didn't really pay much attention. I just knew it was a pill that they took. I had not heard the term ‘aromatase inhibitor’ or Arimidex until I got sick.” (Unfamiliar with AI prior to diagnosis, awareness of tamoxifen)	“Honestly, I come into this with no knowledge when this happened. I arrive at this situation, and they could tell me to eat blue cheese from the moon and I would, you know? I was at their mercy. Whatever they'd tell me, I went, ‘Sure. You have my best interests at heart.‴ (Unfamiliar with endocrine therapies prior to diagnosis) “I knew one lady that was younger, so she was taking the tamoxifen. And just that was part of the deal, I guess. So, I didn't think anything of it… people I have known—after you've had the surgery and the whatever it is, you're going to have that five-year pill as a maintenance.” (Aware of endocrine therapies) “I've known several women who have had breast cancer 20 years ago who went through the whole thing and were put on tamoxifen.” (Awareness of tamoxifen)
Use of online resources for AI-related information gathering	“Very often I get home (from an appointment) and I realize that I don't have the full picture, and so then I go to the Internet.” (Online reinforces information previously received) “The ladies in the group had said that she found the American Cancer site… it is overwhelming, and then I also learned that a lot of the information on there isn’t true, isn’t right.” (Overwhelming volume/ risk for misinformation/inaccuracies) “I got more of that information from reading on the internet than I got from my doctor. I would have liked to have gotten it all from my doctor. She’s part of the process with me. It’s more reliable because you don’t know what you’re reading online.” (Risk for misinformation/inaccuracies; oncologist is preferred source) “I don’t believe I did that (checked online). They say sometimes ignorance is bliss. And I probably just like to stay ignorant so I don’t get all upset about it or worry about it.” (Risk for developing psychosomatic symptoms if checking online) “When Dr. (oncologist) mentioned all of the statistics they just went over my head, but when I see things online, there is retention and I can go back and double check everything so yes I do like- I’m a bit of a cyberchondriac. Just a bit.” (Online reinforces previously received information)	“Before I ever started taking them, yeah I looked for information online. They give us so much information online.” (Online is preferred source, place to gather more information) “I did take my surgeon’s advice, I didn’t go online. There’s too much weird stuff floating around there. A lot of times my daughter will say something and then sometimes I’ll go check it out.” (Overwhelming volume/ risk for misinformation/ inaccuracies) “Well, it's (online) like looking at an encyclopedia. And I mean there are different sites, and you just—it kind of opens my eyes and I realize, hey, I'm not the only one that's going through this. Other people are going through this too… I'm not just an island here … we're a community.” (Online access allows to compare experience with others and validate experience) “I looked up the medication online and I went, “I’ve got that one (side effect). I’ve got that one. I’ve got that one. I’ve got that one.” So this is what’s doing it. So, I’m going to stop now. I think if somebody would have told me, “these are the side effects” that I would’ve stopped it sooner, that I hadn’t waited till I could hardly use my hands and feet.” (Online access allows to compare experience with others and validate experience; as a place to gather detailed side effect information) “Just before I was going to see the doctor, I thought, I’ve got to stop taking this. This isn’t doing me any good. I had her research (that granddaughter got online) what it was doing to my bones and then I made up my mind.” (Online as a place to gather detailed side effect information)
Misconceptions about AI-related symptoms, estrogen, and HRT	“I read somewhere that the more symptoms (side effects) you're experiencing, that's maybe an indicator that the Arimidex is even more effective than it is in folks who may not have any symptoms. And I don't know if that's true. I read that somewhere. But I'm thinking that, okay, I'm suffering like this, but it's for a reason. This medication is doing its job.” (Understanding medication-related side effects link to medication efficacy) “That is, because there's no way to measure it. There's no way to tell what your levels are. ‘Do we know what they're supposed to be? What do you expect of it?’ I would ask the doctor, ‘What are your expectations of a level that would be acceptable?’ It's that unknowing. I'm doing this, but I'm not sure why or if it's doing what I want it to do or they want it to do … I'm an older woman, obviously. I don't know how much estrogen is produced in my body. Do I really need—I mean I realize I do need it, but then, if I totally don't have any—you know what I mean? How is that determination made? Doesn't a person need a certain amount? Am I going to become a little, old man?” (Concerned about whether medication is working, desiring clear lab values, understanding role of estrogen)	“It matters to me to know that I have next to no estrogen in my body. So, why am I blocking it? Why am I taking Advil if I don't have a headache or a sore joint? Why would I take something that offended my body to this huge reaction?” (Desiring clear lab values, weighing up value of endocrine therapies) “Then I looked at the Onco test there with it. I said, ‘Oh. I haven't been paying attention to that estrogen thing.’ I said, ‘I'd better really lose weight.’ I mean this has got to me this week especially. I says, ‘Oh, no. I'm overweight. That's too much estrogen. Without taking the Arimidex, that's too much.’ I should probably take an estrogen test to see how much I have. Is that what they do to evaluate?” (Desiring clear lab values) “In my case, I thought in my age group, that a person—what I didn't know about hormonally, I did not think women at my age had any hormones left. I mean I wasn't aware that they're looming through your body. And I just thought, for younger women, of course, they all, premenopausal, had hormones. After menopause, it's not that big an issue.” (Understanding role of estrogen) “I would see the evidence [Related to seeing a lab-value of estrogen in blood]. I would see the fact that the hormones are changing and the hormones are either going up or down. I would see my personal makeup, and I’d invest in it… if I saw, I would be probably inclined to continue it. In fact, I would still take it. If someone came to me tomorrow and said, ‘Here are your lab results, here is your estrogen level, here's what it can do for you,’ I'd say, ‘Okay. Fine.’ " (Whether or not estrogen levels were high/low enough to warrant endocrine therapy)
Risk perception: Meaning and use of the recurrence statistics	“Well, I try not to read survival statistics because they frighten me, really frighten me. I mean I knew going into my treatments that there was a 50% chance that Herceptin would work and that there was some other percentage that the chemo would work. And altogether, they added up to a 66% cure rate, which is two out of three. And that wasn't quite good enough for me, so I stopped reading statistics because they were frightening… So, you know, I relied on the doctors to guide me.” (Understanding of statistic’s meaning to relative risk: feeling comfort vs feeling fear due to statistics) “I didn’t need to know the odds, I just knew that it would increase my odds of living without cancer.” (Use of recurrence statistics were important) “I was told, ‘if you don’t take it, you may be 3% more likely to have a relapse than your usual percent of relapse’” (Understanding of statistic’s meaning to relative risk)	“The pill makes only a difference of a total of 10%. And that's where I thought, 10% or go through life for the next five years with this (dizziness, bone pain, hot flashes) And I said, “Mm-mm (not interested).” (Understanding statistics meaning to relative risk, compared to quality of life, statistics validate stopping endocrine therapy) “Perhaps if I was told up front, ‘Your skin would dry up, your hands would get more gnarly and more painful because of arthritis,’ I probably would have thought about it a little bit more, wondering whether it was worth it, whether that 11% was worth it. And perhaps I would've tried it just to see.” (Understanding of statistic’s meaning to relative risk) “I know P. (husband) asked at one point what was the percentage of reoccurrence taking medication or not. And he (the oncologist) really couldn't answer me or, you know, come up with any…No. He sort of looked through his paperwork, kind of was doing some calculation. And, of course, P. (husband) was doing his. And, no. Like maybe 10%. I don't know.” (Understanding of statistic’s meaning related to relative risk) “I had like a very small percentage of potentially having another occurrence of cancer. It wasn't nil. It wasn't nil. And then he said, ‘And according to this, I still feel as though you really don't need it. And I've been doing this type of testing,’ for I think it was at that point 20 years. And now he had switched to the Oncotype II testing. He said I had an under 15% chance. (Took comfort in being offered a numerical value of protection associated with the treatment) “The PA kind of suggested—she was the one that said to my daughter it's only a 20% (effective for prevention) …So, when I heard that, that's when I said, ‘Well, it's only 20%,’ and I made my decision. Yeah. It's mainly because my hands were hurting so badly.” (Use of recurrence statistics were important, statistics validate stopping endocrine therapy)

#### Use of online resources for AI-related information gathering

Women in both subgroups used online resources to complement the information they received from friends, peer survivors and their medical team. The majority of women understood the importance of finding a trusted source and mentioned websites such as those of the NIH, Mayo Clinic, American Cancer Society, WebMD, and the website of the treating cancer center. Information obtained online reinforced what they had received from the medical team. Other benefits included more detailed information, explanations of side effects, and the use of online sources as a place to gather information and provide questions to bring to upcoming appointments. Challenges for women in both subgroups using the internet as a source for AI-related information included being overwhelmed by too much information, being concerned about the risk of getting inaccurate and/or impersonal information, feeling confused, getting scared by seeing the worst-case scenarios, encountering medical jargon, and being worried about the potential for developing psychosomatic symptoms. Furthermore, while some women in both subgroups had the ability and access, they chose not to use online sources. The potential for overwhelming, untrustworthy or negative information were the most commonly cited reasons. For example, one participant said, “And I don't think you need to sit on the Internet, because it'll scare the pants off of you.”

Interestingly, more non-persisters reported accessing the internet for AI-related treatment information. In the subgroup of 27 non-persisters, 22 described the internet as an information source, whereas only 14 of the 27 persisters stated they used the internet for AI-related information. However, more women than reflected by these numbers were likely using online information as participants in both subgroups stated a friend or daughter went online, printed information and discussed it with her.

Non-persisters described the internet as a beneficial source of information and support because, though they felt they were struggling alone, the internet allowed them to compare their experience to others. For some, the internet allowed them to know they were not the only woman to have that specific adverse effect or experience. One woman recalled, “(Googling) kind of opens my eyes and I realize, hey, I'm not the only one that's going through this. Other people are going through this too… We're a community.” Non-persisters saw this as a positive aspect of online information sources, given that the majority of the sample did not attend support groups and did not know another breast cancer survivor on an endocrine therapy who had adverse effects and had discontinued. This was dissimilar to how most persisters described the benefits of online sources, which were that online information reinforced what they learned at appointments and assisted in collecting questions and data to ask about at future appointments.

#### Misconceptions about AI-related symptoms, estrogen, and hormone replacement therapies

Women saw side effects as a possibility, yet many did not attribute these negative effects to the AI when they started, especially when the adverse effects started weeks after the AI was first taken or when these effects intensified gradually. Many women from both subgroups also had difficulty disentangling the adverse side effects from typical changes associated with aging. A woman reflected on her thought process related to pinpointing when her side effects started, “I wondered because I’m approaching 70. Maybe this is how people are when they’re 70, they’re suddenly tired.” Not recognizing adverse effects as medication-related meant women did not reach out to their medical team for assistance. For many, this delay led to intense side effects that sometimes escalated to an intolerable point without the assistance of professional help for symptom management.

In general, women did not relate side effects to the lower estrogen resulting from the AI. Likewise, most participants did not connect the symptoms they encountered during menopause and the present estrogen-related side effects at the time of the interview. In addition, the 31% of women who stopped HRT at the time of diagnosis did not consider that abruptly stopping the HRT may have exacerbated their side effects. No woman reported receiving any information on how discontinuing HRT at the time of diagnosis may have impacted their treatment experience.

Women both on and off AIs understood that estrogen level influenced the risk of recurrence and were therefore concerned about whether their estrogen levels were low enough to prevent recurrence. An important difference between the subgroups was that persisters mostly wanted to know about procedures such as scans, x-rays and blood tests they could take to measure their estrogen level to verify the medication was working. In contrast, non-persisters more commonly reported that while they were taking the AI they had never considered whether the medication was working or not. However, some said that since discontinuing, they would want an estrogen level test to see where their levels were without the medication. Non-persisters remained concerned with their risk for recurrence and commented on the importance of providers explaining what comes next for their treatment once they informed the provider they had or would be discontinuing the AI.

#### Risk perception: Meaning and use of recurrence statistics

Although participants were not asked directly about their recurrence statistics, many women injected the topic into the discussion. Women in both subgroups clearly understood that oncologists used statistics in their decisions about recommending endocrine therapy. Accordingly, meaning and use of recurrence statistics were important to women in their decision-making.

The most common source of recurrence statistics referenced by participants was Oncotype DX^®^. While many women took comfort in being offered a numerical value of protection associated with the treatment and agreed that the AI would limit the risk of recurrence, there was considerable misunderstanding in both subgroups regarding how women understood the purpose of statistics and their meaning related to relative risk. For example, some women in both subgroups were confused regarding the Oncotype DX number. Women reported a lack of clarity about their statistical risk for recurrence and recalled various statistics related to their treatment. This confusion was exemplified by a participant who was a professional healthcare provider, still employed full-time, who said, “I am 90% cured, 80% through my surgery and another 10% with taking the Arimidex.”

Interestingly, women in both groups referenced statistics and explained their meaning and value to decision-making related to initiating and continuing endocrine therapy. Nonpersisters also used these statistics to understand and validate stopping endocrine therapy, describing how their percentage of risk for recurrence was “only X%” and how that number was low enough to support the decision to stop. When one woman explained what influenced her decision to stop the AI, she described her physical ailments and limitations and her understanding of the AI’s overall effectiveness stating, “plus the percentage of it working was low, I think it was like 20%.” When side effects started, non-persisters recalled the recurrence statistics they were given and compared the percentage of protection from the AI with what they personally valued in everyday life. Weighing the percentage of protection from the AI with the losses of quality of life in their remaining years, justified and explained the decision to discontinue.

## Discussion

As shown in recent randomized clinical trials targeting AI adherence [17–21], interventions that rely primarily on information strategies do not prevent discontinuation. With an aim to better understand why information strategies do not increase adherence and how they can be improved, here we present the complex and dynamic nuances of gathering and processing treatment-related information found in a sample of 27 women persisting with an AI and 27 intentionally not persisting. While this study was not intended to statistically compare the subgroups, the subjective perspectives bring novel insights for future quantitative research.

Overall, both persisters and non-persisters received and processed information similarly; they reported not having enough trusted information when initiating treatment and when making ongoing decisions regarding adherence. While the impact of prior awareness of AIs on adherence was not measured directly and a causal relationship cannot be stated, several novel findings are of great interest. The first of four misunderstandings or differences between the subgroups was that women who discontinued their endocrine therapies prematurely were more likely to have prior knowledge of AIs. Also, while both groups used online resources, more non-persisters reported doing so. Third, among all participants there were many misconceptions about estrogen’s role, the effects of hormone replacement therapies and challenges in identification of AI-related symptoms. Inconsistency in the meaning of recurrence statistics and their subsequent use in decision-making was evident across subgroups. These results support recommendations to improve patient knowledge and patient-provider communication through clinicians initiating the conversation about AIs at initial and follow-up visits [13]. Our data also show that between information overload, timing of clinical appointments, and changing circumstances, the importance of seeding AI treatment-related information early is essential. The overall quality of information remembered was poor, suggesting the importance of re-visiting information on several occasions, perhaps delivered using multiple methods such as verbal and written take home information. Women wanted more information from their medical oncologist, their most trusted source, which may help clarify confusing information from other sources such as pharmacy inserts, friends, or websites.

In order to provide individualized information, assessing the patient’s personal needs and knowledge is paramount. In accordance with a previous study of women aged 21–80 years [26], our participants knew how much and what type of information they wanted. Therefore, asking their preference early in the relationship could better customize the usual clinical conversation. This discussion might start with asking what information a woman already has about AIs or tamoxifen, including whether she knows someone who received the medication. Conceivably prior awareness about negative peer experiences may set expectations for poor outcomes when women initiate endocrine therapy and pave the way to premature discontinuation. In light of our finding that more non-persisters than persisters reported having prior knowledge of AIs, we urge providers to query women early because patients’ prior knowledge and expectations play a crucial role in adherence [27]. Further research could explore the impact of prior awareness of AIs and tamoxifen on persistence as women aware of AIs may be a higher risk group for discontinuing.

Existing relationships and online resources contributed to and competed with oncologists’ contributions to a woman’s understanding of her treatment. Asking where patients have previously sought information may indicate where they are likely to seek information in the future. In this study, non-persisters were more likely to use online resources, suggesting that women experiencing barriers to persistence such as adverse effects were seeking support and information from sources other than their medical team. A better understanding of how online information is used by older adults is increasingly important [28]. In our study, it was not clear whether women who researched online went to the computer before, during or after they experienced adverse effects of the medications or other barriers to persistence. Prospective research to ascertain at what time point women actively sought informational support online can help the clinician to proactively guide a patient to evaluate the information, how it applies to her individual case, and use the opportunity to discuss management strategies and options.

While self-management of AI side effects is certainly part of post-prescription appointments, those appointments are less frequent than during primary treatment and the opportunity to ask questions and interact with providers are fewer. Participants saw their oncologists as busy and often sought out other trusted resources such as a long-time physician or friend instead of their oncologist. In addition, given the predicted increased shortage of oncologists [29] and the inclination of women to wait until scheduled appointments instead of seeking support between appointments, ensuring women know where to get information moving forward is critical, including when to contact the prescriber if concerns arise. Future research could explore if another professional could fill the medication-support role. Such research can focus on trusted clinicians, such as a primary care provider (PCP) and OB-GYN, and other healthcare professionals, such as oncology nurses and pharmacists, who have been providing care before and during the breast cancer diagnosis. These clinicians, particularly PCPs, may be valuable in providing desired, reliable information and supporting adherence to AIs as the responsibility for follow up care transitions to them in lieu of the medical oncology provider [5]. Researchers in countries other than the US have started to examine how different roles and settings impact adherence. For example, a recent study in Germany revealed that women treated primarily in gynecological practices or in a disease management program had lower risk of treatment discontinuation than those treated only by oncologists [30]. Coordination of care can prevent women from receiving disparate messages from distinct providers. Innovative ways to utilize trustworthy online resources to support women during the post-treatment period is another area for future research.

Women in both subgroups of this sample reported misconceptions about estrogen and HRT. Participants who stopped HRT at the time of diagnosis voiced a need for supplemental information and increased support in side effect management. HRT use prior to and at the time of diagnosis has been noted in the sample characteristics in research conducted in the United States [9, 31] and abroad [32–34]. However, to our knowledge, only one study examined this characteristic and found a significant correlation between HRT use and a higher discontinuation rate of endocrine therapy in the first year after prescription [12]. Future studies with women who stopped HRT at the time of diagnosis are urgently needed.

Women also reported concerns with side effect identification and management. The literature shows a key factor affecting adherence is the experience of adverse side effects that impair quality of life [35]. Therefore, providers need to offer information about and management strategies for symptom control [13]. The importance of asking at each clinical encounter about adverse effects, exploring a woman’s self-management strategies, and expanding her knowledge by offering additional non-pharmacologic tips has been described in women persisting with endocrine therapy [36]. Increased knowledge could lead to improved adherence if an adverse effect is understood better and managed effectively before it becomes intolerable. However, because some side effects may not respond to interventions or, like AI-induced arthralgias, may not have definite effective treatment options [37], communication itself and shared decision-making is an important focus. Another reason ongoing communication about AI-use is important is that the resolve to persist is not a one-time event, but instead is revisited over time [38]. Furthermore, even if a woman eventually stops the medication prematurely, consistent communication about side effects could serve to support and reassure her that she, together with the prescriber, are addressing the adverse effects to the best of their ability. Such demonstration of supportive and collaborative care can serve to motivate women who did not persist with the AI to continue with routine follow-up appointments.

Additionally, the overall impression from the interviews was that participants were surprised to hear about other medication options available when adverse effects started. Awareness of other endocrine therapies before side effects start may encourage women to reach out to the oncologist earlier and consequently support persistence. Another potential contributor to confusion and misinformation was that more women had heard of tamoxifen than an AI prior to initiating the AI and thus based their expectations on what they had heard or read about tamoxifen. It may be valuable for providers to address the two medications and offer an explanation of why they prescribe one endocrine therapy over another.

Finally, discussion about risk and recurrence statistics remains an area for providers to improve clinical communication. Participants in this study attached significant meaning and at times misunderstanding to risk and recurrence statistics such as their Oncotype DX. Some persisters believed that the percentage on their Oncotype DX results was very important and played a central role in initiating and continuing the endocrine therapy, while others described no need to know an exact number, but wanted instead to know that their oncologist recommended an AI and that taking an AI would be protective. For some participants, the scores served as a motivator to start the AI but the same score was also cited as validating the decision to stop when it was later understood to represent low risk. How providers use and explain statistics and genetic technologies are critical in treatment recommendations and women participating in informed decision-making. Thus, further exploration can focus on how to help women optimally use their personal scores and statistics to aide in decision-making. This finding supports a previous study that recommended future research focus on patients’ understanding of this test [39].

Clarifying and tailoring the topics covered in clinical encounters will expand the ability of providers to evaluate many potential factors that may influence adherence and persistence. While side effects play a central role, consideration of a patient’s overall knowledge and health beliefs including her concerns about treatment efficacy and risk all impact the risk for discontinuation and can be explored and supported through enhanced provider-patient relationship. Considering the complex challenges our healthcare system poses to shared decision-making [40], the desire to have patients as full-fledged members of the healthcare team, and for patients and providers to work together to enhance partnership [41], we provide a novel guide based on our findings for providers with short questions to aid in identifying areas of patient’s inaccurate beliefs or unhelpful behaviors (red flags) to promote communication across the AI trajectory in Table 5. These suggested questions translate the results of this study to clinical practice and future research with the intention to move from passive information-giving to more interactive, continuing collaboration to enhance self-efficacy. The clinical communication tips in Table 5 can serve towards an understanding that the need is not always simply more information; instead needs are multi-faceted and require an acknowledgement of the interconnectedness of information and the patient-provider relationship [42]. Thus, these questions aim to support providers and patients in their process of obtaining, managing, understanding and applying information about AIs. Future research of these communication tips should evaluate their use in conjunction and comparison with existing recommendations for clinical communication to further support patient-provider communication and relationship outcomes.

**Table 5:** Keys to guide provider communication with older women receiving an AI.

Provider Goals	Patient Engagement Prompts and Questions Providers Can Ask	Patient Red Flags and *Recommendations for Providers*
Awareness of AIs before prescription		
Patient’s prior knowledge of and potential misconceptions about AIs are assessed and managed as indicated. Patient understands information about AIs is not as common as primary treatments.	Have you ever heard about a pill that some women take for years after they have finished surgery? Have you talked about the AI with anyone who is taking or has taken this type of medication? What did you learn from their experience? Let’s talk about how this person’s experience may be similar to/different than yours.	Patient continues to share inaccurate information. *Provider correct errors and reassess at follow up visits*.
Provider is aware of the AI experiences of other women known to the patient. Provider is aware if patient has AI information that has been gained from various sources including informal networks.	Do you know anyone who took an estrogen inhibitor? How does their experience compare to yours? How long has she been taking it? How is that working for her? Did she have any problems? How did she manage side effects?	Patient continues to share other women’s experiences of non-persistence, non-adherence or death from breast cancer despite adherence. *Provider compares and contrasts the experiences of others indicated by the patient*, *reinforcing positive expected outcome*.
Understanding of how and why the AI is prescribed		
Provider ensures patient is informed of and is able to articulate the role and importance of the AI. Patient verbalizes trust in the treatment plan.	Let’s review your treatment plan and why this medication is important. Let me draw a picture of how the AI works in your body to prevent recurrence. Although you are post-menopausal, you still have estrogen in your body. Sometimes women get information from friends or family, online or from other clinicians that is different from what you got from me. Are you confused about any of the information that we discussed? What is your personal reason for continuing with this pill?	Patient is unable to accurately recall information previously provided. *Provider repeats assessment and information delivery at each follow up appointment*.
Patient clearly verbalizes understanding of her risk of recurrence and the relevance in decision-making.	We just talked about a lot of numbers and statistics. I want to hear from you how you understand their meaning regarding starting/ continuing taking the AI.	Patient shares inaccurate interpretation of recurrence statistics, in specific relationship to her personal risk of recurrence. *Provider restates or re-interprets statistics as indicated*.
Provider assesses and acknowledges patient’s intent to start the AI and corrects information inaccuracies. Provider assists patient in understanding that processing information may take time.	You will start your AI on XXX. Do you have any questions about the dosing schedule?	Patient demonstrates hesitancy or unwillingness to confirm information provided. *Provider restates and verifies patient understands*.
Side effects: Assess informational needs		
Provider is aware the volume of information provided meets the patient’s needs and expectations avoiding information overload Provider acknowledges individual information needs vary. Provider communicates to patient the goal of patient-centered, shared decision-making.	All medications have potential side effects. Some women want to know all the side effects, while others want to know only the most common effects. What is your preference: how much do you want to know before starting?	Patient demonstrates lack of recall of information provided. Patient reports not adhering to dosing schedule. *Provider monitors patient’s understanding and paces delivery and amount of information to match patient feedback*.
Provider shares AI information and assesses sources of information available. Provider acknowledges individual patient learning preferences and identifies and encourages the use of reliable resources for additional information	If you would like, here is some written information about the medication we just talked about. Is it better for you if you have some of this information in writing to take with you? Do you search the internet often for information on AIs or does someone else search for you? Let me give you some reliable medical internet sites I recommend for more information. Another source you can look to for accurate information is the drug package insert and your pharmacist. If you find you have more questions about the AI after you leave this appointment please call or email me.	Patient states incorrect information about AI and declines to indicate the source of the information. *Provider corrects information errors and re-assesses awareness of reliable resources*.
Provider discusses and manages actual and/or potential side effects. Provider conveys understanding of the impact of side effects to the patient. Provider uses collaborative interactive communication in the development of management strategies. Provider is aware of patient’s competing risks such as personal values that risk QOL with potential side effects.	Sometimes side effects begin soon after starting. Some women report they start slowly and later on. Let me know if you experience concerning changes that you may think related to the AI. Please let me know if any of the side effects we have discussed occur so we can move quickly on ways to help manage them. Here are some options for managing this side effect. If the strategy we discussed is not effective in managing that side effect let me know as soon as you can so we can talk about it. I understand you find this side effect difficult to manage. Let’s talk about trying another AI.	Patient continues to mention side effects and denies implementing any recommended interventions. *Provider discusses actual and potential barriers to implementing suggested side effects management interventions*.
HRT stopped at diagnosis		
Provider clearly communicates the relationship between changes in estrogen levels and menopausal symptoms and verifies patient understanding. Provider identifies and discusses potential and actual menopausal symptoms with the patient.	Stopping HRT as you did can result in menopausal symptoms like you may have experienced before you started the HRT. These can be similar to the AI side effects we reviewed. Have you noticed any side effects like those?	Patient expresses surprise or frustration at the return of menopausal symptoms. *Provider reviews cause(s) of side effects and re-introduces management strategies*.
Considering to stop		
Provider is aware of any changeable emotional factors that may influence the patient’s decision-making on stopping the AI.	Are you thinking about suggesting changes to the treatment plan that we initially discussed? Let’s talk about the challenges you are facing with the AI and about some additional strategies. Do you know how to contact my office if you have any questions?	Patient makes statements questioning the necessity of the AI. *Provider engages the patient in discussion regarding the impact of current or anticipated side effects and how those may hinder what she values in her everyday life*.
Deciding to stop		
Provider is aware of and acknowledges any decisional conflict. Provider fosters continued trust in the clinical relationship through support and open communication.	Thank you for sharing your concerns with me. The side effects you talk about sound like they really interfere with the relationships and activities you value. You have told me you are planning to stop /have stopped taking the AI. We have discussed alternatives and I respect that decisions are personal. At this time I want to assure you I will continue monitoring your health and encourage you to maintain our agreed upon follow up schedule. Surveillance is an important part of your survivorship plan of care.	Patient shares information indicating self-blame for stopping the AI. *Provider acknowledges understanding of patient’s difficult decision*, *if indicated*, *and reaffirms provider support and the importance of continuing the clinical relationship*. *Provider assures the patient of the continuation of follow up care*, *including but not limited to the development of a surveillance schedule to assure patient she will be monitored*.

### Strengths and limitations

Strengths of this study are the robust sample size (N = 54) for qualitative inquiry and that women were recruited from both urban and less populated settings in a large geographical area, and received oncology treatment at various medical institutions. The intensive interviews resulted in well-developed, multi-faceted concepts that uniquely highlighted the perspectives of older survivors as opposed to previous research with participants of all ages. Another strength is that methodological and analytical rigor was upheld with an audit trail that included field notes and self-reflective and analytic memos. Trustworthiness was fostered by maintaining the systematic orderliness of data collection and analysis required by constructivist grounded theory methodology that included analytic triangulation through the collaborative effort by the analysis team and checking analytic insights to limit the inherent biases of the researchers.

Constraints of the present study that must be noted for future recruitment are in the sampling. We only included participants who had started AI treatment so these findings do not apply to women who chose not to initiate the treatment. Furthermore, our sample included primarily well-educated Caucasian women (n = 44) with the remaining majority representing East Asian heritage. The above results may not be reliably represented beyond this predominant group, notably missing was representation of Hispanic and African American experiences. While our sample also represensa wide range of ages, marital statuses, and economic backgrounds, a more ethnically and racially diverse group would be a goal for future research teams.

## Supporting information

S1 Table (XLSX)
